# Left Femur Neck Fracture and Left Eye Esotropia as Initial Clinical Presentations of Gastric Cancer in a 54-Year-Old Female Patient: A Case Report and Review of Literature

**DOI:** 10.7759/cureus.20645

**Published:** 2021-12-23

**Authors:** Mohammed S Almasri, Omar N Alfehaid, Ayman Z Azzam, Tarek Amin

**Affiliations:** 1 Department of Surgery, King Faisal Specialist Hospital and Research Centre, Riyadh, SAU; 2 Department of Urology, King Faisal Specialist Hospital and Research Centre, Riyadh, SAU; 3 Department of Surgical Oncology, Oncology Centre, King Faisal Specialist Hospital and Research Centre, Riyadh, SAU; 4 Department of General Surgery, Faculty of Medicine, Alexandria University, Alexandria, EGY

**Keywords:** bone metastasis, medial eye squint, esotropia, pathological fracture, gastric cancer

## Abstract

Although the incidence of gastric cancer has decreased worldwide, it is still among the most common cancers worldwide. Usual manifestations of gastric cancer include gastrointestinal-related symptoms such as weight loss and abdominal pain. Isolated symptomatic bony metastasis at initial presentation is very rare. Here, we present an infrequent case of a middle-aged lady who presented with a left femur neck fracture and left eye esotropia as the initial presenting clinical manifestations of gastric cancer. She was found to have an isolated bone metastasis seen throughout the body skeleton with no extraosseous metastasis. The patient was treated conservatively and received external beam radiation therapy as part of palliative therapy. This report signifies the importance of considering the diagnosis of gastric cancer in middle-aged patients with isolated symptomatic bone metastasis. It also reviews the overall poor prognosis of such cases and recommends further studies on the role of chemotherapy and radiation therapy in such cases.

## Introduction

Gastric cancer is among the most common cancers worldwide, and it was the fourth most common cause of cancer deaths in 2020. According to World Health Organization (WHO), a total of 1.09 million new cases and 769,000 deaths were recorded in 2020 [1]. The most common presenting symptoms of gastric cancer are weight loss and abdominal pain, which are found in more than 50% of all patients [2]. The incident is more common in males than in females, and the mean age of presentation in females is more than 70 years [2]. In a study that included 7,559 patients with gastric cancer, approximately 40% were found to have metastatic lesions. The most common sites of metastasis were the liver (48%), peritoneum (32%), lung (15%), and bone (12%) [3]. However, symptoms of bone metastasis at the time of presentation have been rarely documented and are reported in a few cases in the literature [4-8]. In the current report, we present an infrequent case of a middle-aged lady who presented with a left femur neck fracture and left eye esotropia as the initial presenting clinical manifestations of gastric cancer. An extensive workup was done, which included laboratory workup, X-rays, whole-body bone scan, positron emission tomography-computed tomography (PET-CT), computed tomography (CT), magnetic resonance imaging (MRI) of brain and spine, and upper gastrointestinal (GI) endoscopy, ultimately revealing a primary diagnosis of gastric adenocarcinoma with multiple bone metastasis.

## Case presentation

A 54-year-old female patient was referred to our hospital with chronic pain in the left hip for two months, associated with generalized body ache and easy fatigability. She reported that her left hip pain was getting worse over time without a preceding trauma and is aggravated by physical activities. She had no nausea, vomiting, hematochezia, melena, jaundice, fever, night sweats, or abdominal pain. Her past medical history was significant for type II diabetes only, and her family history was negative for any malignancies. The patient was admitted for further evaluation and workup. The initial laboratory workup results are illustrated in Table 1.

**Table 1:** Initial laboratory workup results of the patient upon her admission.

Laboratory test	Result	Reference range
White blood cells	4.61 10^9/L (normal)	3.90-11.00 10^9/L
Hemoglobin	79 g/L (low)	110-160 g/L
Platelets	164 10^9/L (normal)	155-435 10^9/L
Serum calcium	2.30 mmol/L (normal)	2.10-2.60 mmol/L
Alkaline phosphatase	1,424.0 U/L (high)	46-122 U/L
Cancer antigen (CA) 12-5	12.4 U/mL (normal)	0.0-35.0 U/mL
CA 15-3	12.5 U/mL (normal)	0.0-24.0 U/mL
CA 19-9	110 U/mL (high)	0.0-27.0 U/mL
Alpha-fetoprotein	2.93 U/mL (normal)	0.0-7.0 U/mL
Carcinoembryonic antigen	1.9 ug/L (normal)	0.0-3.4 ug/L

**Figure 1:**
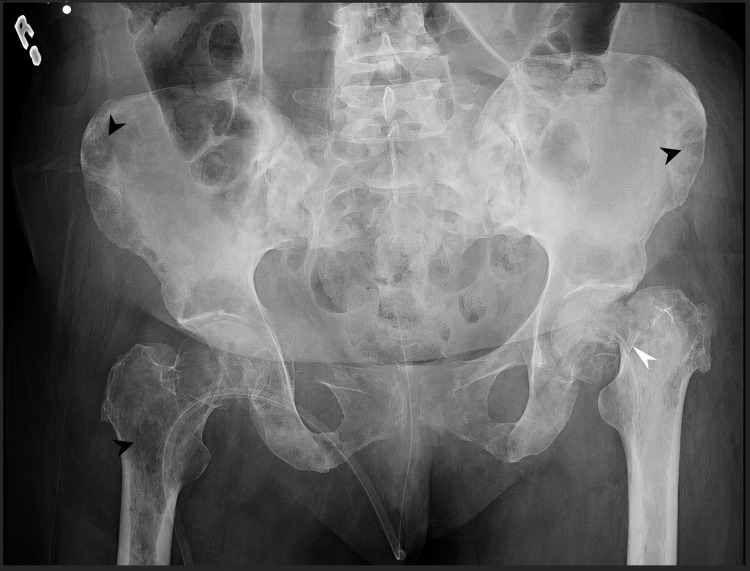
Plain X-ray of the pelvis showing multiple innumerable lytic lesions involving the whole pelvic bones (black arrowhead). A pathological fracture of the left femur neck is noted (white arrowhead).

**Figure 2:**
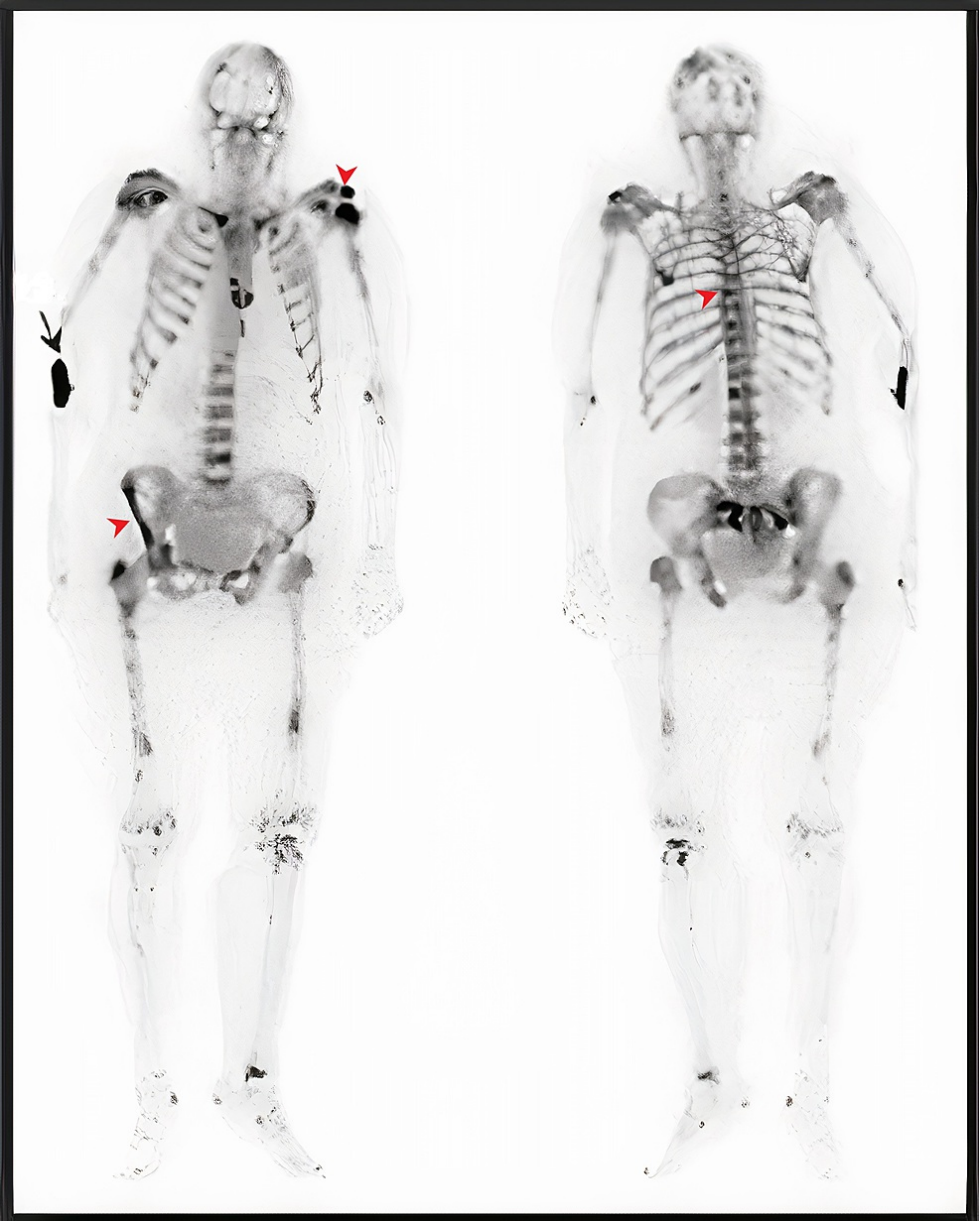
Whole-body scan using single-phase technetium-99-m methylene diphosphonate (MDP) showing extensive MDP avid osseous lesions seen throughout the body skeleton including skull, shoulders, sternum, clavicles, humeri, multilevel spine, pelvic bones, femurs, tibiae, and feet (red arrowhead). The black arrow indicates the isotope injection site.

Upon admission, her left hip pain became intolerable to the point where she could not bear her weight or ambulate alone. On bedside examination, she was found to have a left eye medial squint on general inspection. Her abdomen was soft, not tender, and not distended. Musculoskeletal examination showed shortened and externally rotated left leg. The range of motion in the left hip was extremely limited, and the patient could not abduct or flex her hip. There was tenderness all over the left hip and thigh, but no deformities were noted, and pulses were intact. A plain X-ray of the pelvis revealed a pathological fracture of the left femur neck with incidental multiple lytic lesions involving the whole pelvic bones as seen in Figure 1. A whole-body scan using single-phase technetium-99-m methylene diphosphonate (MDP) was done, which showed extensive MDP uptake seen throughout the body skeleton including skull, shoulders, sternum, clavicles, humeri, multilevel spine, pelvic bones, femurs, tibiae, and feet (Figure 2). PET-CT was done and proved similar findings with no abnormal activity in other systems.

**Figure 3:**
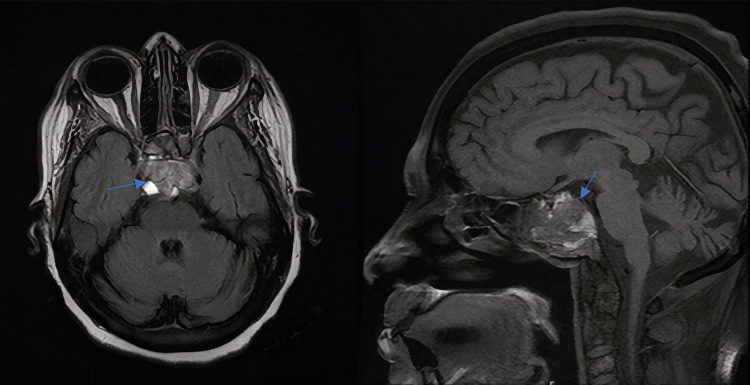
Axial and sagittal views of multiplanar and multisequential brain/orbits MRI with IV gadolinium contrast showing large expansile heterogeneously enhancing complex solid and cystic destructive mass lesion (arrows) involving the central skull base with the destruction of the clivus and extension into the bilateral sphenoid sinuses with no intracranial parenchymal lesions.

As the initial presentation included left eye esotropia, MRI of the brain and spine were done to rule out brain metastasis. MRI showed central skull base mass with multilevel bone marrow replacing lesions in the cervical and thoracic spine (C3, C5, T4, and T5 vertebral bodies) as shown in Figure 3. Multiple myeloma was considered a differential diagnosis, which was excluded by specific laboratory workup. Serum protein electrophoresis showed albumin: 34.80 g/L, alpha-1 globulin: 4.90 g/L, alpha-2 globulin: 6.60 g/L, beta globulin: 8.20 g/L, and gamma globulin: 13.00 g/L. Peripheral blood morphology showed normal WBCs count with no blast or plasma cell noted and RBCs show normocytic blood picture. Ig free light chain kappa was 35.60 mg/L, Ig free light chain lambda was 22.80 mg/L, and kappa/lambda free light chain was 1.561. Urine protein electrophoresis showed predominantly tubular proteinuria.

During her hospital stay, she developed anorexia and multiple episodes of vomiting. The occurrence of those symptoms along with the elevated cancer antigen 19-9 (CA19-9) levels found in the serum at the initial laboratory workup prompted a workup for gastrointestinal malignancy. CT of the chest, abdomen, and pelvis was done, which was unremarkable. Upper GI endoscopy was done and showed a 2 cm ulcerated lesion at the gastric antrum. Multiple biopsies were taken, and the pathology report showed poorly differentiated, diffuse type, signet-ring gastric adenocarcinoma.

The management plan included limb traction, ambulation restriction, and analgesics as needed as part of conservative treatment. Surgical intervention was not an appropriate decision with her extensive disease condition. The patient was prone to multiple other iatrogenic fractures with any surgical intervention including total femur replacement. She eventually received external beam radiation therapy with 08 gray single-shot to skull base metastasis for abducent nerve compression as part of palliative therapy. As there was no rule for any surgical management for her advanced gastric cancer, she was referred to the medical oncology department for further palliative chemotherapy.

## Discussion

Although the incidence of gastric cancer has decreased worldwide, it is still the fourth most common cause of cancer deaths in the world as reported by WHO statistics in 2020 [1]. Generally, gastric cancers are more common in males than in females, with a mean age of 68 years in males and 71 years in females [2]. Our patient here is a female who presents with an advanced stage of gastric cancer at a much younger age (54 years old).

Most of the patients present with GI-related symptoms at initial presentation, which include abdominal pain, weight loss, dysphagia, nausea, vomiting, early satiety, and anemia [2,9]. It is unusual that gastric cancers manifest initially with signs and symptoms of bone metastasis. However, few cases in the literature reported symptoms of back pain [4,6,7], leg pain [5], and double vision [8] as initial manifestations of bone metastasis arising from gastric cancers. Our case manifested with left femur neck fracture and left abducent nerve palsy exhibiting bone metastasis from gastric cancer at initial presentation. A similar case of left abducent nerve palsy was reported by Hirai et al. [8].

Although 85% of reported cases of metastatic gastric cancer to bone also metastasized to visceral organs [10,11], with liver, lung, lymph nodes, and peritoneum being the common sites [3], this case differs as it showed an isolated bone metastasis with no extraosseous metastasis. We also suspected brain metastasis; however, images showed skull base lesions with no intracranial parenchymal lesions. The incidence of skull base metastasis in gastric cancer is extremely rare. There were only four cases reported of skull base metastasis from gastric cancer regardless of initial presentation [8,12-14]. Generally, skull metastasis is considered among the least common sites of bone metastasis in gastric cancers [10]. In our case, skull base metastasis was the likely cause of left eye esotropia. A similar case of abducent nerve palsy of the same diagnosis was reported by Hirai et al. [8]. Another skull base metastasis was reported by Yoshikawa-Kimura et al. [12], mimicking stroke in a patient already diagnosed with gastric cancer.

In the differential diagnosis of such presentation, it is important to include multiple myeloma as differential diagnosis as pattern mimics its clinical manifestation [15,16]. However, the laboratory workup done did not favor the diagnosis of multiple myeloma in this case. With the occurrence of vomiting during the hospital stay, we proceeded with upper endoscopy to rule out GI malignancies.

Patients presenting with bone metastasis are considered to have a poor prognosis. The role of chemotherapy and radiation therapy is still unclear in such cases. Kim et al. performed a cohort study on 1,342 patients with metastatic or recurrent gastric cancer, the initial bone metastasis rate was 6.7%, and the median survival age was 4.4 months after the diagnosis of bone metastasis. In their study, the results supported the conclusion of poor prognosis of such cases; however, the patients who received chemotherapy had a better prognosis as compared to those who did not [17].

## Conclusions

In conclusion, our case report here demonstrates an unusual presentation of gastric adenocarcinoma, which is manifested with isolated symptomatic bone metastasis. Cases presenting with similar characteristics are rarely documented in the literature. The current case report highlights the importance of considering metastatic gastric cancer as a differential diagnosis in patients presenting with pathological fractures and left abducent nerve paralysis as a symptom of extensive bone metastasis. Our review of the literature concluded that similar cases generally have a poor prognosis. Although symptoms can be treated conservatively, no definitive treatment can be offered at this terminal stage. The role of chemotherapy and radiotherapy in such patients is unclear and needs further research.
